# Robotic retroperitoneal lymph node dissection: a stepwise technical guide

**DOI:** 10.1007/s11701-026-03470-x

**Published:** 2026-05-15

**Authors:** Willy Bacaglini, Juan P. Dugarte, Federico Eskenazi, Ignacio A. Bianco, Julio Calderon, Rodrigo Guevara, Arie Carneiro, Gabriel Sibanto, Rene J. Sotelo, Luis G. Medina

**Affiliations:** 1 Division of Urology, Hospital Israelita Albert Einstein, São Paulo, Brazil; 2 Discipline of Urology, Faculdade de Medicina do ABC, Centro Universitário FMABC, Santo André, Brazil; 3 University of Southern California, Los Angeles, CA USA; 4 University of Bologna, Bologna, Italy; 5 Department of Urology, Medical University of South Carolina, Charleston, SC USA

**Keywords:** Robotic surgery, Retroperitoneal lymph node dissection, Testicular cancer, RPLND, Nerve-sparing surgery, Minimally invasive surgery

## Abstract

Retroperitoneal lymph node dissection remains a critical operation in selected patients with testicular cancer, including defined settings of early metastatic disease, post-chemotherapy residual masses, and emerging indications for primary retroperitoneal lymph node dissection in low-volume seminoma. Despite advances in minimally invasive surgery, this operation remains technically demanding because of its proximity to the great vessels, renal hilum, lumbar branches, ureters, and sympathetic neural structures. To present a standardized stepwise technical guide for robotic retroperitoneal lymph node dissection, emphasizing reproducible exposure, peritoneal flap suspension, nerve-aware dissection, posterior plane development, controlled vascular and lymphatic management. This technical report describes robotic retroperitoneal lymph node dissection as a sequential operative workflow. Key steps include patient positioning, port placement, mesenteric incision, creation of a broad L-shaped peritoneal flap, bilateral flap suspension, cephalad stay sutures, entry into the retroperitoneal template, early neural identification, split-and-roll dissection, posterior-first interaortocaval development, and selective clip-based control of vascular and lymphatic branches. The patient is positioned supine in Trendelenburg to facilitate bowel displacement. A broad peritoneal flap is developed and suspended to maintain durable exposure. Dissection proceeds using fixed anatomic landmarks, beginning with controlled access to stable avascular planes. Sympathetic fibers are identified early when nerve preservation is oncologically appropriate. Interaortocaval and para-aortic dissection are performed using a posterior-first strategy to improve orientation and permit deliberate management of lumbar vessels. Mechanical clip control is favored for lymphatic channels and higher-risk vascular branches. Robotic retroperitoneal lymph node dissection can be performed as a structured operation centered on exposure, orientation, neural preservation, posterior plane development, and selective vascular control. Standardization may improve technical clarity during this complex retroperitoneal procedure.

**Supplementary Information:**

The online version contains supplementary material available at 10.1007/s11701-026-03470-x.

## Introduction

Testicular cancer is the most common solid malignancy in young men, and although outcomes are excellent in most settings, retroperitoneal surgery remains important in selected patients. Contemporary guidelines continue to support RPLND in defined clinical scenarios, and the role of surgery has broadened further with renewed interest in primary RPLND for selected low-volume seminoma. This evolution has increased the importance of clear operative standardization rather than diminished it [1–3].

RPLND remains one of the most technically demanding operations in urologic oncology. The procedure requires precise dissection around the vena cava, aorta, renal vessels, iliac vessels, and lumbar branches, while preserving postganglionic sympathetic fibers when anatomically and oncologically appropriate. Open series and post-chemotherapy cohorts continue to show meaningful perioperative burden and nontrivial complication rates, underscoring that technical discipline is central to safe execution [4, 5].

Minimally invasive RPLND has evolved from laparoscopic experience to contemporary robotic series. The robotic platform may facilitate magnified visualization, controlled countertraction, and fine retroperitoneal dissection, but platform alone does not simplify the operation. The critical determinants remain exposure, orientation, template discipline, neural identification, and vascular control. In appropriately selected settings, published robotic experience supports the contemporary relevance of this approach, particularly in experienced centers [6, 7].

The purpose of this article is to present a concise stepwise technical guide to robotic RPLND, with particular emphasis on peritoneal flap suspension, sustained bowel exclusion, nerve-sparing dissection, posterior plane development, and selective control of vascular and lymphatic branches.

## Methods

This report presents a technical description of robotic RPLND as a standardized operative workflow. The procedure is divided into sequential components: patient positioning, robotic and operating room setup, abdominal access, mesenteric incision, development and suspension of the peritoneal flap, entry into the retroperitoneal template, neural identification, split-and-roll dissection, posterior-first template development, and vascular control. The objective of the manuscript is technical and educational rather than comparative.

### Surgical technique

#### Patient positioning and operating room setup

The patient is placed supine on a full-torso gel pad to minimize intraoperative shifting and is secured carefully before docking. A Trendelenburg position of approximately 20 to 30 degrees is used to facilitate cephalad migration of the small bowel. The same target position is maintained even in obese patients to preserve a consistent exposure strategy. The robotic cart is positioned on the patient’s left side, while the assistant or scrub nurse stands on the patient’s right to optimize instrument exchange and workflow.

A standardized arm configuration is used: fenestrated bipolar forceps in the first arm, the camera in the second arm, monopolar scissors in the third arm, and Cadiere forceps in the fourth arm. This arrangement provides reliable traction, dissection, and countertraction during template development. Standard vessel-loop color coding may assist orientation during dissection, with yellow used for nerve fibers, red for arteries, and blue for veins.

#### Access and port placement

Pneumoperitoneum is established at Palmer’s point in the left upper quadrant, approximately 3 cm below the left costal margin at the midclavicular line, using a Veress needle. Port placement is then performed under direct vision to provide central access to the great vessels and sufficient range for contralateral template work.

#### Mesenteric incision and peritoneal flap development

The mesenteric incision begins where the right ureter crosses the right common iliac artery and is extended cephalad toward the ligament of Treitz while avoiding unnecessary dissection near the fixed duodenum. With the fourth arm providing anterior traction, the peritoneal flap is developed posteriorly toward the renal hilum. The flap is kept broad and robust enough to ensure durable bowel retraction. A transverse extension is then carried toward the left side to create an L-shaped opening, which broadens the retroperitoneal working window.

#### Peritoneal flap suspension

After the flap is created, it is suspended with two running sutures on each side using 3 − 0 or 4 − 0 suture. Two bites are taken on each side, and the sutures are secured to the abdominal wall with Hem-o-lok clips. For additional cephalad exposure, two transabdominal stay sutures are placed near the duodenum with two passes to maintain upward traction and stabilize the field. Steady cranial tension is maintained throughout the operation, and additional cephalad suspension sutures are placed as needed. On the left side, traction is initiated early to facilitate preservation of the inferior mesenteric artery.

### Entry into the retroperitoneum and early dissection strategy

The operation uses a split-and-roll technique. A useful early principle is to obtain rapid control of a stable, bloodless plane before progressing through the remainder of the template. In practice, the dissection may be initiated at the vena cava to facilitate control and preserve momentum, while a reproducible anatomic sequence is established from the right common iliac artery toward the aortic bifurcation. This combination of control and fixed landmarks helps preserve orientation during the early stages of the case.

### Caval, iliac, and sympathetic dissection

During lateral and retrocaval dissection, meticulous control of small venous and lymphatic branches is essential to maintain visualization. Hem-o-lok clips are used selectively for these branches. As the dissection progresses, the fibers of the right sympathetic trunk are identified and kept under direct visualization. Once these fibers are recognized, nerve-sparing split-and-roll dissection proceeds in a controlled fashion from the right common iliac artery toward the aortic bifurcation. A vessel loop is used to protect the fibers and facilitate delicate mobilization of the nodal packet.

### Left-sided dissection and renal hilar challenge

On the left side, dissection begins at the common iliac artery. Nerve preservation remains a principle rather than an absolute rule; in some cases, sympathetic fibers course directly into the para-aortic tissue planned for resection and are therefore not preserved. The left renal hilum is often the most challenging area in the supine approach because access is limited and interference can occur between the camera and assistant instruments. During left renal vein and hilar dissection, clips are used to control lymphatics while careful instrument coordination preserves exposure.

### Interaortocaval dissection and posterior-first plane development

Interaortocaval dissection begins with development of the posterior plane. The dissection is directed first toward the posterior abdominal wall and psoas muscle before moving cephalad. This posterior-first strategy improves orientation, facilitates control if bleeding occurs, and allows lumbar vessels to be addressed in a more deliberate sequence. Once the posterior space behind the aorta and vena cava is widely developed, cephalad progression becomes safer and exposure improves substantially. Suction and irrigation are used liberally to maintain a clean field during clearance of interaortocaval and peri-aortic lymphatic tissue.

### Vascular control and contralateral traction

Lumbar arteries and veins are managed in a stepwise fashion. Smaller vessels may be controlled with precise bipolar activation, whereas clips are favored in high-flow regions, especially at the common iliac crossing and the renal hilum. For larger posterior and hilar vessels, mechanical control is preferred over energy alone. After a safe posterior window is created behind the great vessels, the fourth arm is placed posteriorly behind the cava or aorta to deliver steady anterior contralateral traction. This maneuver opens the template, improves exposure, and increases control during deeper lymphatic clearance.

### Completion of the dissection

The final objective is complete template-based clearance with preservation of the right sympathetic trunk in nerve-sparing cases that permit it. The operation is best understood as a sequence built on exposure first, neural identification second, and stepwise vascular and lymphatic control throughout the remainder of the dissection.

## Discussion

The strongest feature of this technique is that it treats exposure as a formal operative step rather than an intermittent problem. A robust L-shaped peritoneal flap, bilateral running suspension sutures, and cephalad stay sutures near the duodenum convert bowel management into a planned part of the operation. This matters because RPLND becomes more difficult not only when anatomy is complex, but when the field becomes unstable. In that sense, the flap is not merely a retraction maneuver; it is a foundational step that permits the rest of the dissection to remain structured.

A second key principle is early sympathetic trunk identification and nerve-aware split-and-roll dissection. In RPLND, preservation of ejaculatory function has long been one of the major technical goals when oncologically appropriate, and refined dissection depends on identifying the relevant fibers before nodal mobilization obscures the plane. A vessel-loop-assisted split-and-roll sequence under direct vision helps keep the dissection deliberate rather than reactive. Current guideline-era management continues to support the relevance of RPLND in selected settings, making careful attention to nerve-preserving technique clinically meaningful rather than purely academic [1].

The posterior-first interaortocaval strategy is equally important. By developing the deep plane toward the psoas and spine before aggressive cephalad progression, the surgeon gains earlier control of lumbar branches and a more stable sense of the posterior boundary. This is especially valuable in an operation where bleeding from posterior vessels can rapidly degrade visualization. Published open and post-chemotherapy series reinforce that RPLND remains a consequential operation with meaningful complication risk, which is precisely why sequence, exposure, and vascular control deserve standardization [4, 5].

The robotic platform may support these objectives through magnified visualization, articulated dissection, and stable fourth-arm countertraction. Prior laparoscopic literature established the minimally invasive foundation for template-based retroperitoneal dissection, while more recent robotic multicenter data suggest that robotic RPLND has an expanding role in experienced hands. Still, the value of the present manuscript is not to claim superiority of robotics over open or laparoscopic surgery. Its value lies in translating a complex retroperitoneal operation into a reproducible technical sequence centered on exposure, orientation, and controlled progression [6, 7].

## Conclusions

Robotic RPLND can be approached as a standardized stepwise operation centered on stable exposure, robust peritoneal flap suspension, early neural identification, nerve-sparing split-and-roll dissection, posterior-first template development, and selective clip-based vascular control. A disciplined operative sequence may improve technical clarity during this complex retroperitoneal procedure.

## Supplementary Information

Below is the link to the electronic supplementary material.

Supplementary Material 1

## Data Availability

No datasets were generated or analysed during the current study.
